# A Case of Profound Bradycardia in Endurance Athlete with Severe Anorexia Nervosa

**DOI:** 10.1155/2022/6589758

**Published:** 2022-11-07

**Authors:** John Pueringer, Joseph Cinderella, M. Greg Treuth

**Affiliations:** ^1^Philadelphia College of Osteopathic Medicine, Philadelphia, Pennsylvania, USA; ^2^TidalHealth, Salisbury, Maryland, USA

## Abstract

We present a case of a 54-year-old woman with asymptomatic bradycardia who was referred for consideration of a pacemaker for profound chronic sinus bradycardia (heart rate is 33 beats per minute). Further, history and physical revealed a self-described endurance athlete with severe anorexia nervosa (AN). *Background*. Anorexia nervosa and endurance training are known contributors to bradycardia; however, profound asymptomatic sinus bradycardia in the 20-30 beats per minute is underdocumented in the literature and not a common presentation in any setting. The decision to implant a permanent pacemaker could potentially lead to further physical and psychological repercussions in such a frail patient.

## 1. Case Report

A 54-year-old female is presented to the emergency department (ED) with altered mental status. The patient had a history of anorexia nervosa (AN), treated with Mirtazapine and Hashimoto's thyroiditis, for which she took levothyroxine. The patient was compliant with medications. A week prior, she had a seizure-like episode attributed to hypoglycemia. Her admission blood glucose was 24 mg/dl. This was felt to be the etiology of her altered mental status, and following administration of 50% dextrose, she became responsive. Computed tomography (CT) scan of the head was unremarkable. The patient was found to be hypothermic at 89 degrees F, for which she was placed on bear hugger. She was markedly cachectic, weighting 88 pounds with a body mass index (BMI) of 14.12. The patient was a self-described endurance athlete and claimed to workout 4 hours per day, 7 days per week. She also followed a calorie deficient diet, which was evidenced by her extensive muscle wasting and cachectic state. In addition, she had chronic sinus bradycardia noted on previous records. She denied any purging or laxative use and was under the care of a psychiatrist for the AN; however, her condition has not improved much over the last few months. The patient was admitted for unexplained fugue, profound bradycardia, hypoglycemia, and hypothermia.

**Figure 1:**
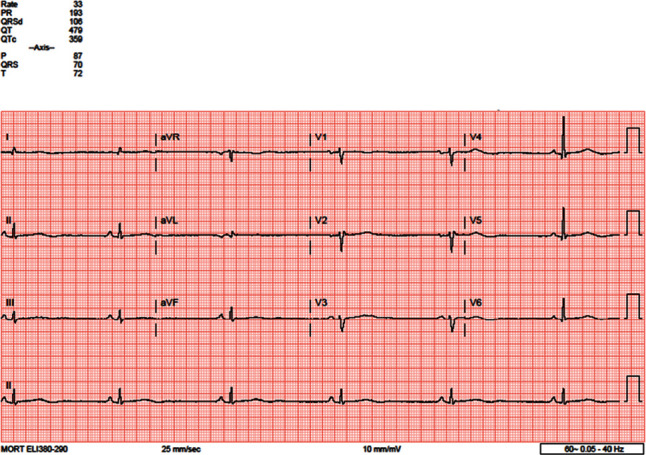
ECG from 11/2021 showing sinus bradycardia of 33 bpm.

**Figure 2:**
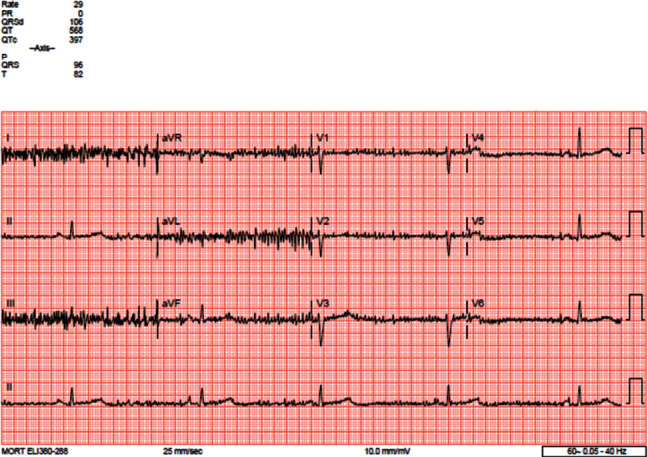
ECG from 10/2019 showing sinus bradycardia of 29 bpm.

**Figure 3:**
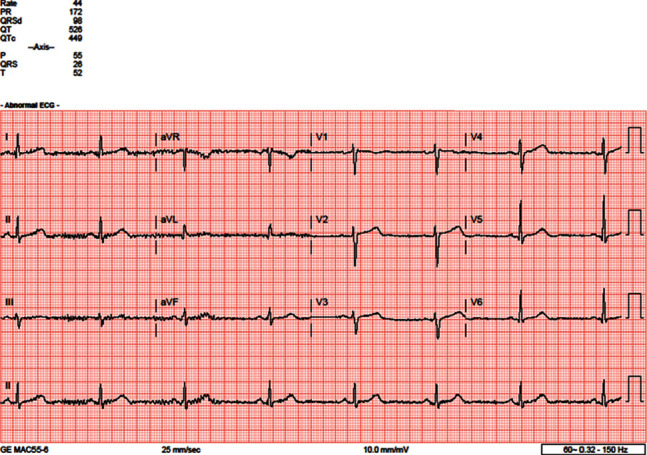
ECG from 09/2017 showing sinus bradycardia of 44 bpm.

Cardiology was consulted regarding heart rate dipping into the 30 s; the thought, being this, could potentially also account for her altered mentation. Electrocardiogram (EKG) showed pronounced sinus bradycardia at 33 beats per minute (Figure 1). A week earlier, EKG in the ED showed sinus bradycardia at 36 beats per minute. In October 2019, EKG showed sinus bradycardia at 29 beats per minute (Figure 2). The oldest EKG noted was 2017, with a sinus bradycardia at 44 beats per minute (Figure 3). A Holter monitor had been ordered in the past, but the patient never carried it through. CT scan of the head showed no acute intracranial abnormality. Transthoracic echocardiogram revealed an ejection fraction (EF) of 55% but was otherwise unremarkable. Exercise tolerance test (ETT) was performed to look for chronotropic incompetence; however, there was no evidence to suggest chronotropic intolerance. The stress test was a regular “Bruce Protocol” ETT. She went 5′50^″^ and achieved a heart rate of 141 beats per minute, and the test was reportedly stopped due to fatigue. She reached 7 metabolic equivalents (METs). The study was read and reported that her heart rate accelerated as expected. She also reportedly achieved 85% of her predicted maximum heart rate (PMHR). Chest radiograph was unremarkable. Workup for adrenal insufficiency was negative. She did have a cortisol level of 30.1 mcg/dl and a prolactin level of 6.0 ng/dl; both of which are normal. Female sex hormone or growth hormone levels were not obtained.

## 2. Treatment

The patient was placed on telemetry on admission with no sign of heart block as evidenced on the EKG in Figure 1. The presumptive explanation for her symptoms was her profound hypoglycemia. She had Hashimoto's thyroiditis, although current labs revealed, essentially, the euthyroid state (Table 1). Electrophysiology (EP) was consulted for evaluation of treatment of sinus bradycardia with possible pacemaker implantation. Exercise tolerance test showed no evidence of chronotropic intolerance. In the absence of symptoms, ventricular arrhythmia or structural heart disease, and owing to normal ventricular function, no permanent pacemaker implantation was undertaken. The etiology of her chronic sinus bradycardia is likely multifactorial but related to her AN. The heart rate increased to 60 s during her stay. Refeeding was gradual to prevent refeeding syndrome and further heart damage. The patient would continue treatment with her psychiatric team in the outpatient setting, who currently had her on Mirtazapine 7.5 mg daily, with no current plans for a permanent pacemaker. A Holter monitor was considered but not done due to her past medical history of noncompliance. She was discharged home with arrangements for a life alert bracelet, glucose monitoring devices, and glucose tablets.

## 3. Case Discussion

Anorexia nervosa (AN) is a psychiatric disorder characterized by low body weight (e.g, BMI < 18.5 kg/m^2^) caused by disordered energy intake relative to requirements. It can also be secondary to endocrine and metabolic changes in the body. Anorexia nervosa has the highest mortality rate of any of the psychiatric disorders [1]. Weight loss and chronic malnutrition lead to dysfunction in multiple organ systems, notably cardiac dysfunction, where sudden cardiac death accounts for 60% of deaths associated with the disorder [2].

There are many documented cardiovascular complications of anorexia, including bradycardia, dilated cardiomyopathy, electrolyte-induced arrhythmias, hypotension, mitral valve prolapse, and pericardial effusion [3]. Anorexia nervosa can affect the structure and size of the heart by causing left ventricular atrophy and sometimes annular changes leading to mitral valve prolapse, possibly secondary to reduced blood volume [2]. An autopsy study showed left ventricular atrophy with endocardial and interstitial fibrosis in a patient with AN [4]. It is plausible that bradycardia may be the hearts physiologic response to myocardial atrophy to prevent heart failure.

Effects of anorexia on the electrical activity of the heart include a heightened vagal tone in the setting of substantial weight loss. The mechanism of bradycardia in AN is thought to be due to cardiac vagal hyperactivity and decreased metabolism of energy utilization, due to low caloric intake [5]. It was reported that patient with AN had a 30% increase in vagal activity [5, 6]. A resting heart rate of less than 50 bpm was found in 70% of patients in a consecutive series [7]; however, most of the individuals were under the age of 25, compared to our 54-year-old patient. Furthermore, as we present here, a resting heart rate of 20-30 beats per minute is not as well documented. Patients may also present with prolonged QT_c_ interval secondary to electrolyte disturbances (such as hypokalemia, hypomagnesemia, and hypophosphatemia), which is thought to be the root of sudden cardiac death in patients with AN [1].

Anorexia can cause alterations in cardiac conduction and repolarization and thus result in a range of arrythmias. Sinus bradycardia has been described as the most common cardiovascular physical finding and the most common arrhythmia in patients with anorexia nervosa [7].

Resting bradycardia is a known physiologic change in endurance athletes. Studies have shown that endurance training is able to enhance vagal control of the heart and to reduce the resting HR by more than 8% in older adults who trained for more than 30 weeks [8]. Interestingly, D'Souza et al. posit that training-induced bradycardia may be the result of widespread remodeling of pacemaker ion channels, notably a downregulation of funny channel HCN4 and the corresponding ionic current [9]. In addition, the HERITAGE family study found that after 20 weeks of endurance training, resting heart rate is reduced by approximately 5 bpm, with greater reductions occurring in female than males [10]. Sinus bradycardia and first-degree atrioventricular block are common arrhythmias documented in endurance athletes, among other [11]. Therefore, it is probable that her aerobic training and AN had an additive effect on her vagal hyperactivity, or possible synergistic effect, although that would need to be studied further. However, her athletic training was self-described as a rigorous 4 hour, 7 days-a-week running schedule, but there was no real data in her chart that she participated in any sports or athletics other than her self-described running.

It is important to consider what led a 54-year-old female athlete to anorexia nervosa. She claimed to be physically active, although not well supported on chart review. We do not think she was an athlete who somehow decided to become anorexic. It would appear that her primary issue was anorexia, and running helped her maintain what she felt was a desirable body weight. We would argue that she was not in particularly good shape for a runner, as documented by her limited functional capacity on the stress test. However, the importance of her proclaimed 28-hour per week training schedule is that it parallels prolific endurance athletes who typically perform 15 to 40 hours of training per week. Cycling, triathlon, cross-country skiing, rowing, and endurance running are examples of sports in which intensive training has been associated with the highest exercise capacities and the most profound cardiac remodeling [12]. Thus, any history of this prolific endurance training is likely contributing to her cachectic anorexic state in addition to exacerbating her presenting arrythmia through chronic remodeling and enhanced vagal tone. We notably did not consult psychiatry, because our patient was being treated in the outpatient setting with Mirtazapine 7.5 mg. On further chart review, our patient was unable to tolerate Fluoxetine and Sertraline due to side effects. In addition, she had a history of denying or creating excuses to eat or get intravenous nutrition when hospitalized for recurrent syncope, hypoglycemia, and unresponsive states. She understood that she had anorexia nervosa but minimized and denied the connection between her recurrent symptoms and her urgent medical status. Therefore, it would appear that her resistance to proper nutrition may be the primary culprit. Of note, Mirtazapine can cause orthostatic hypotension, hyponatremia, serotonin syndrome, increased QTc interval, and torsades de pointes, which is relevant in this patient with these symptoms and workup for syncope. Furthermore, bradycardia increases the risk for torsades de pointes. The combination of AN-induced bradycardia and Mirtazapine could put her at increased risk for torsades de pointes. However, based on her history of present illness and clinical findings, we do not feel like this influenced her syncopal episode.

A study by Mehler and Brown reported bradycardia in greater than 95% of anorexic patients, and hypotension likewise is quite common [13]. Our patient had both. The article then goes on to state that many of these patients attribute their bradycardia to what they perceive as an “athletic heart” and often attempt exercise to maintain or lose weight. Our patient was put on a treadmill to document chronotropic competence and avoid a pacemaker. She only went 5 minutes and 50 seconds on a Bruce protocol treadmill before stopping due to fatigue. That protocol would be 2.5 miles per hour (mph) at a 12-degree incline, hardly what an “athlete” would be capable of. The average person her age can go approximately 9 minutes, and an athlete should be able to go 12-15 minutes. Her systolic blood pressure (SBP) during the treadmill stress test remained in the 85-105 mmHg range, consistent with the chronic hypotension of anorexia. This posits that the bradycardia is related to heightened parasympathetic tone, as the body tries to conserve energy due to limited energy reserves from chronic starvation.

Low energy availability is a relatively common complaint among female athletes. Energy availability is defined as the difference between energy obtained through oral nutrition and energy expended with exercise. It is reasonable to conclude that low energy may result as a consequence of increased energy expenditure, decreased oral nourishment, or both. Chronic energy deficit in the female athlete can result in musculoskeletal and reproductive dysfunction. Low energy in combination with menstrual disorder and altered mineral bone density is known as the classic “female athlete triad”. In individual cases, the patient may require multidisciplinary care from an endocrinologist, an orthopedic surgeon, a psychiatrist, an exercise physiologist, and in some cases, a cardiologist. Many female athletes, however, fail to maintain adequate energy intake. When this energy deficit is intentional, it is described as disordered eating. The energy deficit in anorexia nervosa causes a heightened parasympathetic tone that is part of the body's compensatory mechanism to conserve energy when faced with limited caloric reserves. Bradycardia and hypotension are the consequences.

Our indication for “treatment” would be a recognition that bradycardia is very common in anorexia, and understanding that pacing is rarely or never required. The most common indications for permanent pacemaker implantation are sinus node dysfunction (SND) and high-grade atrioventricular (AV) block [14]. For our patient, the absence of symptoms, ventricular arrhythmia or structural heart disease, and owing to normal ventricular function, we felt that permanent pacemaker implantation was unnecessary. According to the American College of Cardiology (ACC), the American Heart Association (AHA), and the Heart Rhythm Society (HRS) Guidelines on the Evaluation and Management of Patients with Bradycardia, in patients with sinus node dysfunction with minimal or infrequent symptoms without hemodynamic compromise, temporary transcutaneous or transvenous pacing should not be performed [15]. Furthermore, in asymptomatic patients with sinus bradycardia or sinus pauses that are secondary to physiologically elevated parasympathetic tone, permanent pacing should not be performed. Thus, unless the patient shows symptomatic bradycardia, a pacemaker is generally not indicated.

Accordingly, treatment should be directed at the underlying psychiatric issues and not for unnecessary pacemaker or other procedures. The stress test was done to confirm chronotropic competence, or the ability to appropriately raise one's heart rate with activity. Alternatively, a Holter monitor or another monitor could have been done. The problem with relying on a monitor is the difficulty in teasing out bradycardia as the cause of her recurrent near syncopal spells. In retrospect, the syncopal spells appear to be due to anorexia associated hypoglycemia, but confirming a lack cardiac syncope in light of the concerns related to her bradycardia.

Other possible etiologies of syncopal episodes and sinus bradycardia could be underlying heart disease, heart block, toxic ingestion of medication, electrolyte derangements (hyperkalemia), hypothyroidism, hypothermia, hypoxemia, hypovolemic shock, infection, and intracranial hypertension, but there was no clinical or lab evidence of this, and the echocardiogram revealed no structural or functional abnormalities to her heart. The EKG was able to rule out heart block or any underlying arrythmias.

Additionally, since this patient showed up bradycardic, hypoglycemic, hypotensive, mild hyponatremic, and with a syncopal episode, there was a concern for thyroid dysfunction as the etiology. She was diagnosed 3 months ago, initially with subclinical hypothyroid, but the patient was compliant with meds and in a euthyroid state. She was treated with Levothyroxine 50 mcg and 0.5 mg of Dexamethasone, and we also ordered an adrenocorticotropic hormone (ACTH) stimulation test, which was benign, helping us rule out adrenal insufficiency. The patient did present in a hypothermic state, but correction of her temperature did not affect her underlying heart rhythm. The patient did not present with a hypoxemic or hypovolemic picture.

## 4. Conclusions

Anorexia nervosa can cause profound sinus bradycardia due to cardiac vagal hyperactive and decreased caloric intake. In the presence of asymptomatic bradycardia secondary to severe anorexia nervosa, an implantable pacemaker did not appear necessary as it may cause more physical and psychological harm than good. Severe anorexia in combination with long-term aerobic training may have a synergistic effect on vagal tone and its effects on resting heart rate; however, that needs to be studied further.

## Figures and Tables

**Table 1:** Notable vitals and laboratory values.

Vitals		Chemistries		CBC		Endocrinology	
Temp	89.6 F	Na	133 mEq/L	WBC	5.5/mm3	TSH	8.5 mU/L
Heart rate	33 bpm	K	3.7 mEq/L	Hb	12.5 g/dl	T3, free	1.9 ng/dl
Respiratory rate	10-14 bpm	Cl	99 mEq/L	Hct	36.20%	Free T4	1.12 ng/dl
Blood pressure	94-99/73-78	CO2	24 mEq/L	Plt	203/mm3	Cortisol	30.1 mcg/dl
Sp02	100%	BUN	47 mg/dl			Prolactin	6.0 ng/dl
POCT Glucose	47 mg/dl	Cr	0.75 mg/dl				
BMI	14.12	Glu	24 mg/dl				
		Calcium	8.3 mg/dl				
		Mg	2.1 mg/dl				
		ALT	81 U/L				
		AST	65 U/L				
		ALP	43 U/L				
		Bili total	0.6 mg/dl				
		Troponin	0.03 ng/ml				
